# Awareness of Breast Cancer among Female Students at Ain Shams University, Egypt

**DOI:** 10.5539/gjhs.v6n1p154

**Published:** 2013-11-04

**Authors:** Dina N. K. Boulos, Ramy R. Ghali

**Affiliations:** 1 Community, Environmental and Occupational Medicine Department, Ain Shams University, Cairo, Egypt; 2 Clinical Oncology Department, Ain Shams University, Cairo, Egypt

**Keywords:** awareness, breast cancer, female university students

## Abstract

The present study aimed to determine knowledge of breast cancer risk factors, symptoms and early detection methods and to identify knowledge and practice of breast self-examination among Ain Shams University female students. This is a descriptive cross sectional study. Most study participants had low level of knowledge of breast cancer risk factors. The most widely known risk factors by the students were smoking 66.9%, followed by radiation to the chest 63.7% and genetic factors 63.7%. Most of the students (81.6%) identified breast lump as a symptom for breast cancer. However, non lump symptoms were less known and less than half were aware of other warning signs. Mass media such as TV and/or radio were identified as the main source of information on breast cancer by 89.1% of students followed by relatives 39.2%. Only 8.8% of students identified correctly the appropriate time to perform breast self examination and 1.3% reported performing it regularly every month. The most common reasons for not practicing BSE were” did not know how to perform it” (47.7%) and lack of interest (35%). The findings of this study showed that there is low level of knowledge on breast cancer risk factors, early warning signs and BSE among female university students and that only few students practice BSE monthly. Health care workers should develop effective breast health programs targeting youth to help females to gain healthy habits starting very early during their formative years.

## 1. Introduction

Breast cancer is the most commonly diagnosed cancer and worldwide it is considered the leading cause of cancer death in females, accounting for 23% (1.38 million) of the total new cancer cases and 14% (458,400) of the total cancer deaths in 2008. Approximately half of the breast cancer cases and 60% of the deaths are estimated to take place in developing countries (Ferlay et al., 2008).

According to the official statistics of the Egyptian National Cancer Institute, breast cancer represents 18.9% of total cancer cases (35.1% in women and 2.2% in men) (Elatar, 2001). In Egypt, the median age at diagnosis for breast cancer is ten years younger than in the United States and Europe (Omar et al., 2003). Cancer in young is generally more aggressive and results in lower survival rates, making early detection even more crucial and emphasizing the importance to raise breast cancer awareness among young females (Rosenberg & Levy- Schwartz, 2003; Sambanje & Mafuvadze, 2012). Breast cancer mortality rates for African women are higher compared to women in Western countries (Kamangar, Dores, & Anderson, 2006)

Although the importance of Breast Self Examination (BSE) is controversial (Hakama, Pukkala, Kallio, Godenhjelm, & Svinhufvud, 1995; Thomas et al., 2002), the American Cancer Society recommends it for early detection of breast cancer as it assists women in two main ways; first by becoming familiar with both the appearance and the sense of their breasts and second by helping them to detect any changes in their breasts as soon as possible (American Cancer Society, 2008).

Although there were scarcity of studies on knowledge of breast cancer and practice of BSE among Egyptian females (Allam & Abd Elaziz., 2012), to our knowledge there were no studies about the knowledge of breast cancer and practice of BSE among Egyptian female university students.

The present study aimed to determine knowledge of breast cancer risk factors, symptoms and early detection methods and to identify knowledge and practice of breast self-examination among Ain Shams University female students.

## 2. Methods

### 2.1 Study Design

This is a descriptive cross sectional study.

### 2.2 Study Population

Undergraduate students at non health related disciplines were included based on a belief that they possess less health information compared to the general public. The study sample included 543 female students at non health related disciplines, who were available on the day of data collection, who agreed to participate in the study and who returned a completely filled questionnaires (Response rate=89.8%).

### 2.3 Study Tool

A self administered questionnaire in Arabic language was distributed by fourth year medical students to study participants in order to clarify any item if needed A Clinical oncology consultant evaluated content and face validity of the questionnaire. Moreover, the questionnaire was pilot tested on 20 students (not included in study sample) to check the clarity of the questions. Results of the pilot test were used to adjust the wording of some questions in order to make them easier to understand. The Cronbach's alpha of study questionnaire is 0.805. The questionnaire covered the following items

#### 2.3.1 Part one:

Socio-demographic data such as age, marital status, discipline, academic year.

#### 2.3.2 Part two:

- Knowledge of risk factors for breast cancer was determined with 13 questions. The answers were "true", "false" and "don’t know". This part assessed the knowledge of breast cancer risk factors using the guidelines of the American Cancer Society (2008)
- Knowledge of early warning signs, of methods of early detection and of different lines of treatment of breast cancer

#### 2.3.3 Part three:

- Knowledge of Breast Self Examination (BSE) term and correct time to perform it
- Practicing BSE and reasons for not performing
- Source of information on breast cancer

### 2.4 Ethical Consideration

Approval of study conduction was obtained from the ethical review Committee at the faculty of Medicine Ain Shams University. In addition, the purpose of the study was explained to all participants and confidentiality was assured, an oral informed consent was obtained and the survey tool was anonymous.

### 2.5 Data Management and Statistical Analysis

Data collected were revised, coded and computerized. Data entry using Statistical Package for Social Science (SPSS) version 18 was used. Frequency tables and a chart were utilized to describe nominal variables. Continuous variables were described using distributions, means and standard deviation.

## 3. Results

### 3.1 Characteristics of Study Participants

Participants in this study ranged in age from 17 to 23 years with a mean of 19.3 (SD=1.1). Of 543 participants 43.1% were students at commerce school. More than one third of study participants 36.7% were second year faculty students, 31.7% were fourth year and 25.7% were third year and only 5.9% were first year faculty students. The majority of participants 90.4% were single. Only 16.1% of students reported a family history of breast cancer. Nearly all study participants 98.7% believe that early detection improves treatment outcome and as many as 87.7% believe that there is an effective treatment for breast Cancer (Table 1).

**Table 1:** Sample characteristics

Characteristics	Frequency	
	N=543	Percent
**Age in years**		
Mean ± SD	19.26±1.1	
Range	(17-23)	
**Faculty**		
Literature	146	26.9
Commerce	234	43.1
Law	55	10.1
Languages	108	19.9
**Faculty year**		
First	32	5.9
Second	99	36.7
Third	140	25.7
Fourth	172	31.7
**Marital status**		
Single	491	90.4
Engaged	44	8.1
Married	8	1.5
**Family history of Breast Cancer**		
Yes	88	16.2
No	397	73.1
Do not know	58	10.7
**Believe that early detection improves treatment outcome**		
Yes	536	98.7
No	1	0.2
Do not Know	6	1.1
**Believe that there is an effective treatment for Breast Cancer**		
Yes	477	87.7
No	20	3.7
Do not Know	46	8.5

### 3.2 Knowledge of Risk Factors

Most students had low level of knowledge of breast cancer risk factors. The most widely known risk factors by the students were smoking 66.9%, radiation to the chest 63.7% and genetic factors 63.7%. Age at first full term pregnancy >30 years and never being pregnant were not known as risk factors for breast cancer by most of the students (Table 2).

**Table 2:** Students’ Knowledge of breast cancer risk factors

Risk Factors	True		False		Do not know	
	N	%	N	%	N	%
Aging	140	25.8	225	41.4	178	32.8
High Fat Diet	197	36.3	112	20.6	234	43.1
Obesity	124	22.8	176	32.4	243	44.8
Smoking	363	66.9	67	12.3	113	20.8
Radiation to the chest	346	63.7	38	7	159	29.3
Alcohol	246	45.3	75	13.8	222	40.9
Oral contraceptive use	105	19.3	126	23.2	312	57.5
Never breast fed	111	20.4	201	37.1	231	42.5
Family history of breast cancer	258	47.5	157	28.9	128	23.6
Genetic Factors	346	63.7	48	8.8	149	27.5
Never being pregnant	38	6.9	286	52.6	219	40.3
Age at first full term pregnancy > 30years	32	5.9	201	37	310	57.1
Early menarche (<12 years)	40	7.4	160	29.4	343	63.2
Late menopause (>55 years)

### 3.3 Knowledge of Symptoms, Early Detection Measures and Lines of Treatment

Most of the students 81.6% identified breast lump as a symptom for breast cancer. However, non lump symptoms were less known and less than half were aware of other warning signs such as change in shape/or retraction of nipple and bloody nipple discharge accounting for 25.6% and 24.7% respectively. Further, as many as 74.2% of students identified breast self examination as an early detection measure for breast cancer. The most widely known lines of treatment were surgery followed by chemotherapy accounting for 71.8% and 67.7% respectively (Table 3).

**Table 3:** Knowledge of warning signs, early detection and treatment of Breast cancer

Variables	True		False		Do not know	
	N	%	N	%	N	%
**Warning signs of breast cancer**						
Breast lump	443	81.6	12	2.2	88	16.2
Bloody nipple discharge	134	24.7	61	11.2	348	64.1
Pain in breast	403	74.2	55	10.1	85	15.7
Change in shape and/or breast size	281	51.7	64	11.8	198	36.5
Redness of breast skin	189	34.8	61	11.2	293	54
Change in shape and/or retraction of nipple	139	25.6	42	7.7	362	66.7
**Early detection measures**						
Breast self examination	403	74.2	44	8.1	96	17.7
Mammogram	283	52.1	31	5.7	229	42.2
Breast U/S	262	48.3	36	6.6	245	45.1
**Treatment of Breast Cancer**						
Surgery	390	71.8	50	9.2	103	19
Radiotherapy	180	33.1	113	20.8	250	40.04
Chemotherapy	368	67.7	60	11.1	115	21.2
Hormonal therapy	94	17.3	118	21.7	331	61
Targeted therapy	88	16.2	79	14.6	376	69.2

### 3.4 Source of Health Information

**Figure 1:**
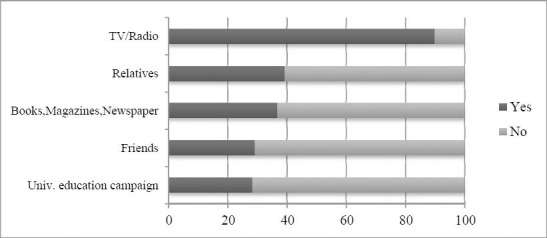
Sources of students’ information

Mass media such as TV and/or radio were identified as the main source of information on breast cancer by 89.1% of students followed by relatives 39.2% (Figure1).

### 3.4 Breast Self Examination

More than half of the students (63.4%) heard about BSE. Only 8.8% of students identified correctly the appropriate time to perform BSE. The percentage of students performing BSE regularly every month was 1.3% and 6.1% reported that they performed it irregularly. The most common reasons for not practicing BSE were “did not know how to perform it” (47.7%) and lack of interest (35%) (Table 4).

**Table 4:** Students’ knowledge, performing and reasons for not performing BSE

Variables	N	%
**Heard about BSE**		
Yes	344	63.4
No	199	36.6
**Knowledge of appropriate time for BSE**		
Correct answer	344	8.8
Incorrect Answer	75	13.8
Do not know	420	77.4
**Performing BSE**		
No	503	92.6
Regular	7	1.3
Irregular	33	6.1
**Reason for not doing BSE**		
Do not know how to perform	240	47.7
Fear of positive finding	37	7.4
Forgetting	30	5.9
Not interested	176	35
Not sure of its ability in detection	20	4

## 4. Discussion

Informing youth about breast cancer is both a challenge and key investment in the health of future generations of women (Gürsoy et al., 2009) as it is well known that low cancer awareness contributes to delays in presentation of cancer symptoms, and subsequent diagnosis leading to less favorable outcomes (Nystrom, 2000).

In the current study more than half of the students (63.4%) heard about BSE. This study showed that female university students may not have adequate knowledge about BSE as only 8.8% of the students had knowledge about appropriate time to perform BSE. Our findings are comparable to a study of Yemeni University students in which 76.9% of participants heard about BSE (Ahmed, 2010). The current study findings are lower than the following studies; Iheanacho, Ndu and Emenike (2013) in which 36% of female undergraduate Nigerian students have knowledge on the appropriate time for BSE, Budden (1995) study revealing that 77% of the female students correctly identified the recommended time for BSE and Milaat (2000) reporting that 7.1% of secondary-school female nursing students had had knowledge about appropriate time for BSE. A cross-sectional study that included 718 female high school students in Turkey showed that only 37.9% of the students reported to hear about BSE and 13.2 % provided correct response for its appropriate time (Karayur, Özmen, & Çetinkaya, 2008).

In the current study only 1.3% practice BSE regularly every month and 6.1% reported that they performed it irregularly. Our findings are lower than (Karayur et al., 2008) study in which 6.7% of high school students in Turkey were performing BSE monthly and 20.3% of the students were performing BSE irregularly. In other studies the percentage of monthly BSE performance have been found to be 3.4% among teenagers (Ludwick & Gaczkowski, 2001), 17.4% among female Yemeni university students (Ahmed, 2010), 13% of the first year of nursing students (Budden, 1999), 14.8% among students aged 17 to 30 years in Europe (Wardle et al., 1995), 37% among female university nursing students in Australia (Budden, 1995). The above mentioned studies revealed the low prevalence of youth monthly performing BSE all over the world.

Studies from Egypt have revealed that the percentage of older women performing BSE monthly ranged from 2.7% to 18.8 % (Abdel Fattah et al., 2000; El Shamy & Shoma, 2010).

It is anticipated that a higher percentage of women in older age groups perform BSE because they are at higher risk of breast cancer. However, studies from Egypt have shown low percentages of both young and older women performing BSE. This may be due to the fact that health education programs organized to increase breast health awareness are not satisfactory. Such education programs should start very early in the formative years.

In the present study mass media such as TV and/or radio were identified as the main source of information on breast cancer by 89.1% of students followed by relatives 39.2% and 36.8% acknowledged books, magazines and newspapers as the leading source of information. This is consistent with Iheanacho et al. (2013) study showing that majority of the students had acquired information about breast cancer, its risk factors and BSE from sources such as media (68%), from books (68%), school (lectures, friends) (66%) and from health care providers (64%).

Similarly media was acknowledged as the main source of information on breast cancer by 48.6% of female high school students in Turkey while 44.4% of the sample mentioned health professionals as a source of information (Karayur et al., 2008). In another study of Chinese women aged 20 years or greater mass media, such as newspaper and television, was acknowledged by 73.2% as the major information source on breast cancer, followed by doctors or health care providers as identified by 16.1% (Yan, 2009). Likewise (81.6 %) of Yemeni university students mentioned mass media as the first source of information about breast cancer (Ahmed, 2010). These findings indicate that media continue to be one of the most important sources of information about breast cancer and BSE.

Not knowing how to perform BSE was the primary reason for not practicing BSE as reported by 47.7% of Nigerian female students, lack of interest was identified by 35% and only 7.4% mentioned fear of a positive finding as a reason (Iheanacho et al., 2013). This agrees with a Yemeni study in which 55.9% of students mentioned lack of knowledge about technique of BSE as a barrier for not practicing BSE (Ahmed, 2010). Similarly the most common reasons for not doing BSE stated by Turkish high school students were "not knowing how to perform BSE" (98.5%), "not expecting to get breast cancer" (45.6%) and "fear of discovering a breast lump" (8.8%) (Karayur et al., 2008).

In the current study nearly all study participants (98.7%) believe that early detection improves treatment outcome and as many as 87.7% believe that there is an effective treatment for breast Cancer. However, most students had poor knowledge of breast cancer risk factors. The most widely known risk factors by the students were smoking (66.9%), radiation to the chest (63.7%), genetic factors (63.7%) and Family history of breast cancer (47.5%). Age at first full term pregnancy >30 years and never being pregnant were not known as risk factors for breast cancer by most of the study participants.

Similarly a study carried out to determine the awareness of breast cancer risk factors and practice of breast self- examination among female students of the University of Nigeria Enugu Campus showed that the only risk factors that are widely known are family history of breast cancer (50%), and tobacco smoking (36%). The findings also showed that very few students had knowledge of other risk factors (obesity, alcohol intake, early menarche, nulliparity, breastfeeding for at least 18 months as possible risk factors for breast cancer (Iheanacho et al., 2013).

Likewise the most widely known risk factors by Turkish high school students were personal history of breast cancer (68.7%) and family history of breast cancer (67.0%). In other words, students know that breast cancer was associated with genetic factors (Karayur et al., 2008).

Most of the students (81.6%) in the present study identified breast lump as a symptom for breast cancer followed by pain in breast as reported by 74.2%. However they were less knowledgeable about non lump symptoms and less than half were aware of other warning signs such as change in shape/or retraction of nipple and bloody nipple discharge accounting for 25.6% and 24.7% respectively. Further, as many as 74.2% of students identified breast self- examination as an early detection measure for breast cancer. The most widely known lines of treatment were surgery followed by chemotherapy accounting for 71.8% and 67.7% respectively.

In concordance with the current study a cross-sectional survey of Angola university students using a self-administered questionnaire, showed that almost all study participants (80%) reported that breast lumps that are cancerous would be painful (Sambanje & Mafuvadze, 2005). Powe et al. (2005) reported that association of pain with cancer is a common myth and in fact pain is not necessarily a warning sign of breast cancer as many persons associate pain with the occurrence of cancer and the fact is pain is not necessarily an early sign of breast cancer.

Knowledge of symptoms and methods of diagnosis of breast cancer was mostly above (60%)among nurses working in a general hospital in Lagos, Nigeria and the most widely reported symptom identified by(93.6%) of the m was breast lump (Odusanya & Tayo, 2001).

## 5. Study Limitations

The present research is as a cross sectional study on female university students in one of the most eminent governmental universities in the capital of Egypt as a result it does not represent Egyptian female youth population as a whole also the results of the current study do not represent the entire population of Egyptian female university students all over Egypt. In addition, the study participants were relatively homogenous as a group so sociodemographic comparisons were not performed.

## 6. Conclusion

Health behaviors acquired early in life have an influence on future health. The findings of this study showed that there is low level of knowledge on breast cancer risk factors, early warning signs and BSE among female Ain Shams University students and that only few students practice BSE monthly. There is a need to raise the knowledge of university students about the risks of breast cancer and benefits of early detection. Health care workers should develop effective breast health programs targeting youth to help females to gain healthy habits starting very early during their formative years.
